# Lipophilicity Modulations by Fluorination Correlate with Membrane Partitioning

**DOI:** 10.1002/anie.202301077

**Published:** 2023-04-17

**Authors:** Zhong Wang, Hannah R. Felstead, Robert I. Troup, Bruno Linclau, Philip T. F. Williamson

**Affiliations:** ^1^ School of Chemistry University of Southampton Highfield Southampton SO17 1BJ UK; ^2^ Department of Organic and Macromolecular Chemistry Ghent University Campus Sterre Krijgslaan 281-S4 9000 Ghent Belgium; ^3^ Centre for Biological Sciences University of Southampton Southampton SO17 1BJ UK

**Keywords:** Aliphatic Compounds, Fluorination, Lipophilicity, Membrane Partitioning, NMR Spectroscopy

## Abstract

Bioactive compounds generally need to cross membranes to arrive at their site of action. The octanol‐water partition coefficient (lipophilicity, log*P*_OW_) has proven to be an excellent proxy for membrane permeability. In modern drug discovery, log*P*_OW_ and bioactivity are optimized simultaneously, for which fluorination is one of the relevant strategies. The question arises as to which extent the often subtle log*P* modifications resulting from different aliphatic fluorine‐motif introductions also lead to concomitant membrane permeability changes, given the difference in molecular environment between octanol and (anisotropic) membranes. It was found that for a given compound class, there is excellent correlation between log*P*_OW_ values with the corresponding membrane molar partitioning coefficients (log*K*_p_); a study enabled by novel solid‐state ^19^F NMR MAS methodology using lipid vesicles. Our results show that the factors that cause modulation of octanol‐water partition coefficients similarly affect membrane permeability.

## Graphical abstract

A novel solid‐state ^19^F MAS NMR method was developed to accurately and easily measure water‐membrane partitioning values (*K*_p_). Given the very different octanol and membrane environments, a remarkable correlation between log*K*_p_ and log*P* was found, but only for a given compound series. This work indicates that even minor lipophilicity modulations by aliphatic fluorination can have true relevance for the drug discovery optimization process.

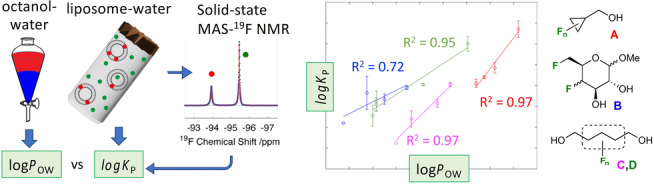

The lipophilicity of a compound has been shown to be an excellent proxy for a host of physical parameters related to ADMET (absorption, distribution, metabolism, excretion and toxicity).^[1]^ It relates to a compound's ability to partition into the cell membrane, and as such it provides a valuable metric to describe the ability of a pharmaceutical to permeate the various cell membranes required to access its site of action.[^1^, ^2^] Lipophilicity is measured as the octanol/water partition coefficient (log*P*_ow_) of a compound, or by indirect methods based on correlations with chromatographic retention times.^[3]^

Despite the success of lipophilicity as a parameter in the drug discovery process, the octanol phase is clearly a drastically simplified model for the complex composition and chemical environment of the cell membrane.^[4]^ In contrast to the isotropic octanol environment, the anisotropic cell membrane presents a highly complex environment composed of many lipid species each with their own physicochemical properties that determine the spatial organization and dynamics of the lipids within the lipid bilayer.^[5]^ To reflect these differences, correlation studies between log*P*_OW_ and the membrane molar partition coefficient (*K*_P_), a unitless value describing the ratio of the mole fractions of a compound in bilayer to that in the aqueous phase, have been carried out.^[6]^ Although these studies show a general correlation between log*P*_OW_ and partitioning in lipid vesicles (log*K*_P_), significant variations are observed.^[7]^ These differences arise due to the differences in the physicochemical properties of octanol and the cell membrane that influence their interaction with the drug.[^4^, ^5^, ^8^] Typically compounds show a higher affinity for the cell membrane than for octanol, with the magnitude of these discrepancies depending on enthalpic and entropic contributions to the partitioning.^[9]^ This is explained by charged species being well tolerated in the high‐dielectric lipid headgroup region. In addition, steric effects contribute, with octanol accommodating bulky groups that would otherwise disrupt the packing of the lipids within the bilayer.^[4b]^

Fluorination of drug candidates is a much‐employed strategy to modify both biological and physical properties of drug candidates, and the resulting impact on log*P*_OW_ can be significant. Hence, much research has been carried out in investigating how fluorination motifs modulate lipophilicity.^[10]^ However, to the best of our knowledge, a detailed study on the influence of fluorination on water‐membrane partitioning is not available. Zhang et al. have determined the *K*_p_ values of trifluoroethanol, hexafluoro‐2‐propanol and nonafluoro‐*t*‐butanol using fluorescence quench methods as part of a study of their bilayer modifying properties.^[11]^ While not specifically mentioned, a qualitative agreement between log*P* (clog*P* in the case of C_4_F_9_COH) with log*K*_p_ is apparent. Hence, this calls for further research regarding the effect of aliphatic fluorination on membrane permeability.

The extensive recent research into aliphatic lipophilicity prompted us to investigate whether the often small log*P* modifications caused by subtle changes in fluorination substitution are actually replicated in membrane partitioning, or in other words, whether 1‐octanol is still a valid model for membrane permeability when considering purely aliphatic fluorination modifications. For this purpose, closely related compound analogues with subtly different fluorination motifs are employed, using aqueous solutions of multilamellar lipid vesicles composed of 1‐palmitoyl‐2‐oleoyl‐glycero‐3‐phosphocholine (POPC) and a mixture of POPC/cholesterol as a mimic of the eukaryotic cell membrane. Mindful of membrane anisotropy and steric factors, three different compound series **A**–**C** (Figure 1) with varying steric demands and lipophilicity range (1.49 log*P* units) were selected. The cyclopropyl methyl compounds and the carbohydrate derivatives are models for relatively rigid compounds, whereas the 1,5‐pentanediol derivatives are models for conformationally flexible compounds.

**Figure 1:**
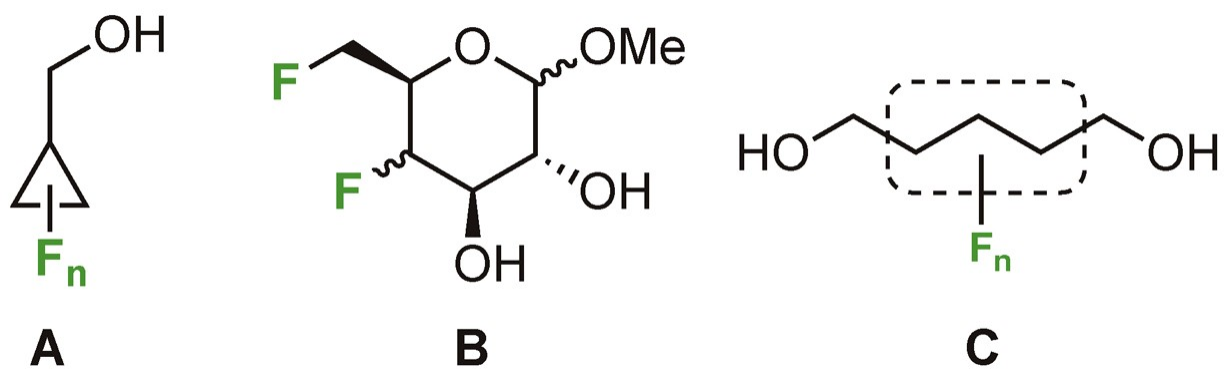
Compounds used in this study.

We also describe the development of convenient magic‐angle spinning (MAS) ^19^F solid‐state NMR methodology to determine the partitioning of these (non‐UV‐active) fluorinated compounds into lipid vesicles without the need for separation of the vesicles. Solution‐state ^1^H and ^19^F NMR methods for *K*_p_ measurement have been developed that rely on the detection of the free compound in solution by exploiting perturbations in resonance position, linewidth and/or T_1_ relaxation arising from the rapid exchange between the free and membrane bound states.^[12]^ Typically this necessitates titration across a range of lipid concentrations, making such an approach labour intensive and time consuming, but it benefits from not relying on accurate quantitation of the concentration of the compounds in the free and membrane bound state.[^4a^, ^8^] NMR techniques that are able to measure compounds partitioned into a membrane are more challenging to use, as the restricted motion in a membrane environment leads to a broadening of the line‐shape due to the chemical shielding anisotropy, susceptibility effects and dipolar couplings present. While solid‐state NMR methods are widely used to determine the localisation of drugs within a cell membrane,^[13]^ its application for the determination of log*K*_p_ is more limited. A ^2^H solid‐state NMR method has been developed for log*K*_p_ determination, where both the free and bound deuterated species can be analysed. Partitioning of deuterated molecules into membranes results in the formation of the broad quadrupolar Pake pattern in the ^2^H NMR spectrum, which can be deconvoluted from the isotropic signal arising from the free compound, allowing the ratio of free to membrane bound compound to be determined.^[14]^ Nevertheless, this method requires isotope labelling whilst quantitation is challenging as the signal from the motionally restricted deuterium spectrum which is low in intensity and distributed across a broad range of frequencies must be integrated against the sharp intense peak of the free compound.

Given this work deals with investigating the effects of fluorination on membrane partitioning, we considered ^19^F solid‐state NMR methodology, in which the exquisite sensitivity of the ^19^F chemical shift to changes in electrostatic environment would be exploited.^[15]^ We postulated that application of magic‐angle spinning with proton decoupling would sufficiently average the anisotropic nuclear spin interactions that become apparent in the spectrum upon partitioning in the membrane to obtain baseline‐separated resonances of the compound in water and in the lipid environment. The absence of fluorine in naturally occurring lipids further simplifies any spectrum, eliminating any spectral ambiguity and facilitating the accurate integration of the resonances necessary for the calculation of the molar partition coefficient *K*_P_.

The principle of the method is explained in Figure 2 (with a detailed description of the protocol provided in Supporting Information). The method utilizes ^19^F MAS NMR to quantitate the partitioning of the fluorinated compounds between the aqueous and membrane phases. To our delight, two resolved resonances were observed, which not only shows that the sensitivity of the ^19^F resonance to the local environment is able to distinguish between the compound in the aqueous and in the membrane phase, but also that exchange between the aqueous and membrane pools is slow on the NMR timescale, i.e. $k_{exch}\ll \Delta \delta$ (for a detailed discussion see Supporting Information). Experimentally, it was determined that only moderate spinning speeds (10 kHz) and low power decoupling (10 kHz decoupling field) were required to obtain well resolved spectra, even at these high magnetic fields; with motional processes within the liquid crystalline lipid bilayer averaging the potentially large chemical shielding anisotropy and dipolar couplings experienced by the fluorine spins. The application of low spinning speeds and radiofrequency decoupling fields also reduces sample heating, whilst minimising the pelleting of the vesicles against the rotor wall. The absence of any discernable sidebands, resulting from incomplete averaging, simplifies the analysis, as this precludes the need to integrate the entire family of sidebands.

**Figure 2:**
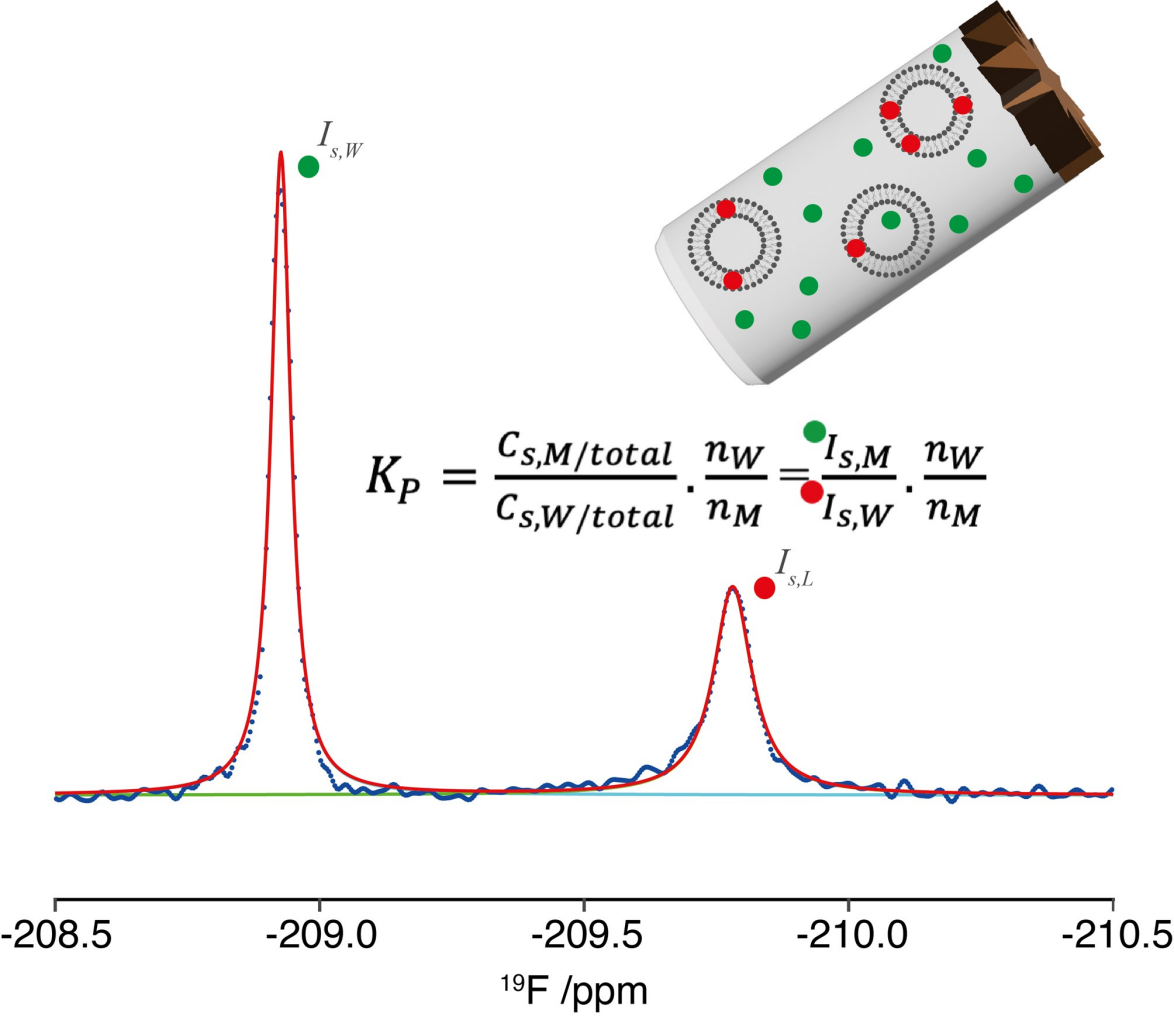
Schematic explaining ^19^F MAS NMR (850 MHz) is utilized to calculate molar partition coefficient ($K_{P}$ ). ^19^F MAS NMR spectra of fluorinated compounds exhibit two resonances arising from the populations in the water (W) and membrane phases (M), whose intensities ($I_{s,W})$ and ($I_{s,M}$ ) reflect the concentrations ($C_{s,W}$ and $C_{s,M}$ ) of the compounds in each environment. With knowledge of the number of water ($n_{W}$ ) and lipid ($n_{M}$ ) molecules, the molar partition coefficient ($K_{P})$ is readily determined.

To determine how the the concentration of the compound and bilayer hydration influenced the measured log*K*_P_, a systematic study of these parameters was undertaken for compound **10** (Figure S2, Table S1). Samples were prepared where the lipids were hydrated at levels between 10 to 30 %(w/v), a range widely used to study the physical properties of lipid bilayers. This resulted in a mean log*K*_P_ of 1.85±0.06 across the range of compound concentrations studied. Similarly, variation of compound concentration from 1 to 10 mol % (with respect to total lipid) showed similar reproducibility with a mean log*K*_P_ of 1.85±0.02 across all levels of hydration. On the basis of the reproducibility of these figures, all subsequent experiments were conducted at 20 % (w/v) hydration with a compound/lipid ratio of 0.033. In addition to providing excellent reproducibility, these concentrations afforded good signal to noise ratios with sensible spectral acquisition times.

The structures of the studied compounds and the obtained log*K*_p_ values are shown in Figure 3. The corresponding log*P*_OW_ values are provided in purple, with a range of 0.36 log*P* units for the cyclopropyl derivatives,^[16]^ and 0.54 and 0.81 log*P* units for the carbohydrate and 1,5‐pentanediol^[17]^ derivatives. Overall, the correlation between the log*K*_p_ and log*P*_OW_ values (Figure S3) is only moderate, with a correlation coefficient of 0.59. However, when each series is considered individually, excellent log*K*_p_–log*P*_OW_ correlations are obtained (Figure 4).

**Figure 3:**
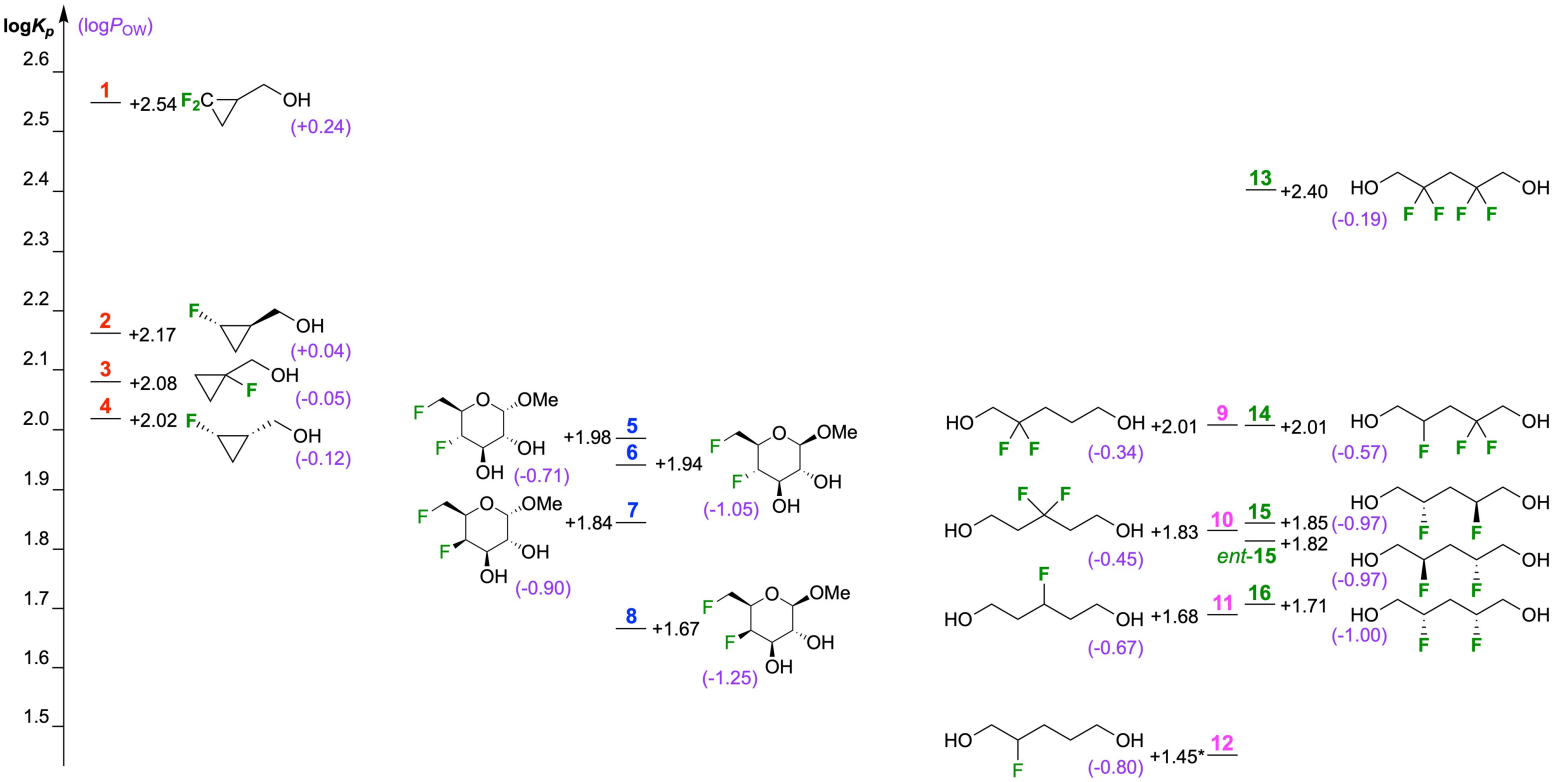
The structures of the compounds used, with their log*K*_p_ values (values reported are a mean of 3 independent experiments). Octanol‐water lipophilicities are shown in blue.

**Figure 4:**
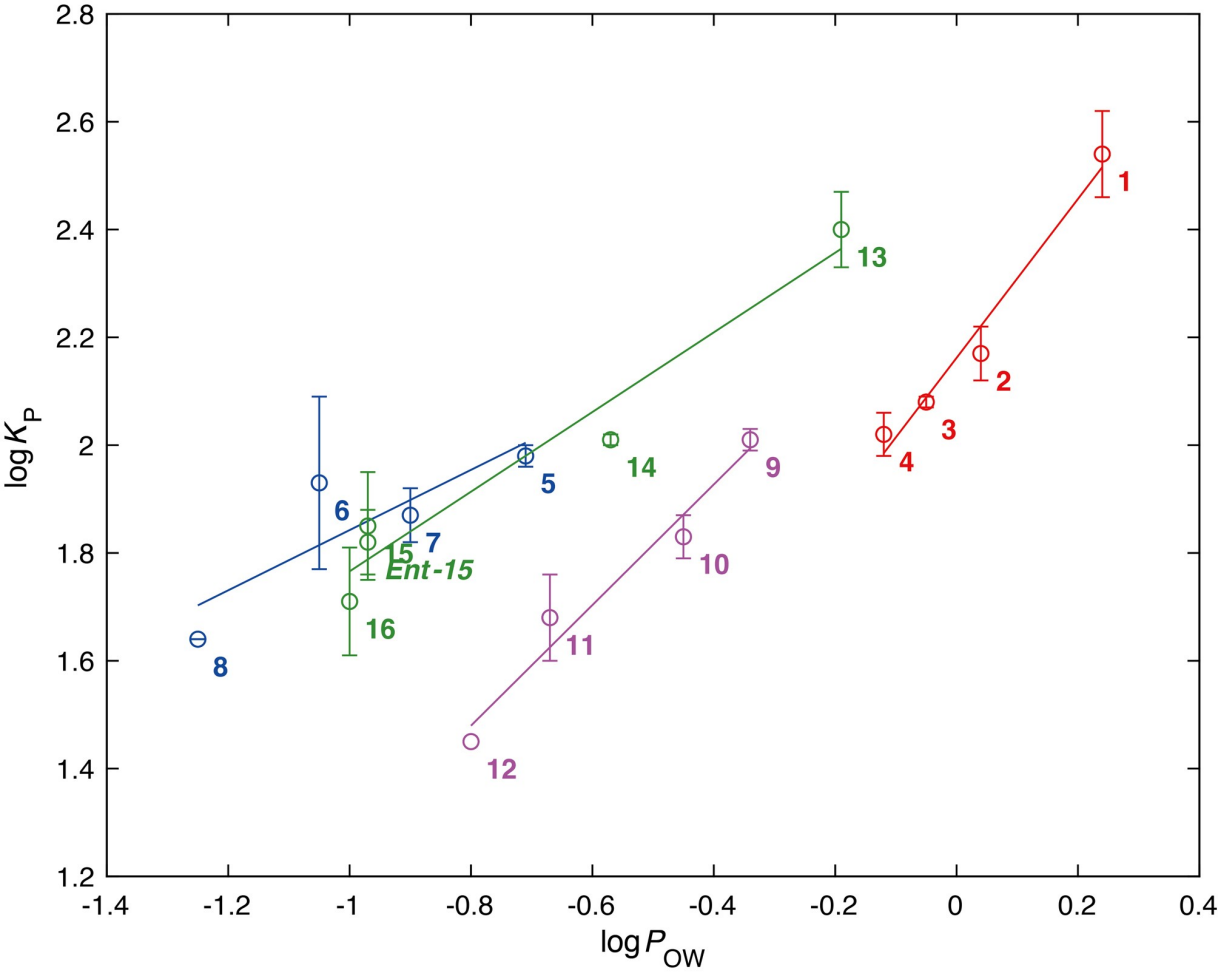
log*K*_p_/log*P*_OW_ correlation for each series of compounds. The labelling and the structures of each compound is the same as for Figure 3.

For the cyclopropylmethyl derivatives **1**–**4**, the log*K*_p_–log*P*_OW_ correlation (*r* ^2^=0.97) includes comparisons between substrates having different relative fluorine stereochemistries (**2** vs **4**), fluorination position (**2**,**4** vs **3**), and number of fluorines on a given carbon atom (**2**,**4** vs **1**). For the glycosides **5**–**8**, which also involve changes at a non‐fluorine containing stereocentre, log*P* changes between epimers are reproduced in the corresponding log*K*_p_ values (**5** vs **6**; **7** vs **8**; **5** vs **7**; **6** vs **8**), but this is not the case when two stereocentres are inverted simultaneously: the methyl‐β‐4,6‐difluorinated glucoside derivative **6** has a lower log*P* value compared to methyl‐α‐4,6‐difluorinated galactoside derivative **7**, but a higher log*K*_p_ value. This results in a moderate log*K*_p_–log*P* correlation (*r* ^2^=0.72). For the more flexible pentane‐1,5‐diol derivatives, excellent log*K*_p_–log*P*_OW_ correlations were also obtained when two separate series were considered: compounds **9**–**12** with fluorination at a single position (*r* ^2^=0.97), and compounds **13**–**16** with a skipped fluorination motif (*r* ^2^=0.95). For both series, lipophilicity differences arising from positional, fluorination number and diastereochemical changes are nicely reflected in their corresponding log*K*_p_ values. An interesting case concerns the enantiomers **15**/*ent‐* **15**. A fundamental difference between 1‐octanol and POPC vesicles (or membranes) is that the former is achiral, while the latter are composed of enantiomerically pure constituents. By definition, enantiomers have identical log*P* values, but could be expected to have different log*K*_p_ values. The measured log*K*_p_ values for **15** and *ent*‐**15** however, are virtually identical, and within error of each other.

It is interesting to note that there is an apparent difference in the ability of octanol/water and liposome systems to discriminate between the lipophilicity, as captured by the slope of the correlations (Figures 4 and S4). For the glycosides **5**–**8**, log*P*_OW_ provides greater discrimination with a slope of 0.56, whilst the lipophilicity of cyclopropyl derivatives are best discriminated by the liposome based model, with a slope of 1.47. Surprisingly, even the two very similar series of pentane‐1,5‐diols **9**–**12** with a fluorination and a single carbon and compounds **13**–**16** with a skipped fluorination motif exhibited differing lipophilicity dependencies with slopes of 1.20 and 0.74 respectively. Such variations have been explained by the relative ability of the two hydrophobic phases to accommodate incoming molecules, with the “malleable” octanol environment and the more structured bilayer environment responding differently to molecular shape and range of interactions.^[4]^

Cellular membranes exhibit significant variations in lipid composition, a property that is also not reflected in octanol/water partition experiments. A major advantage of the method presented here compared to log*P* determination is that it can be applied to membranes of arbitrary complexity, enabling the analysis of the influence of such properties as headgroup size/charge and chain length/saturation. As naturally occurring lipids are devoid of fluorine and do not contribute to spectral complexity, our solid state ^19^F NMR MAS methodology is readily extended to natural lipid extracts, and in principle, with appropriate attention paid to data interpretation, to intact cells. As an example of this kind of investigation, a mixed lipid system was employed. In particular, we investigated how increasing concentrations of cholesterol, a commonly found sterol in eukaryotic cell membranes, influenced the membrane partitioning of **9** and **10** (Figure 5, Table S3). In contrast to pure POPC membranes, increasing levels of cholesterol up to 50 mol %, a level found in native membranes,^[18]^ resulted in a reduction in the partitioning of the compound into the membrane, with the log*K*_P_ of **9** and **10** falling by 0.24 and 0.17 respectively. The fall in log*K*_P_ mirrors earlier studies where a reduction in membrane partitioning for small molecules has been observed.^[19]^ This reduction has been attributed to the disruption of the acyl chain packing and their favourable interactions with the cholesterol.^[20]^

**Figure 5:**
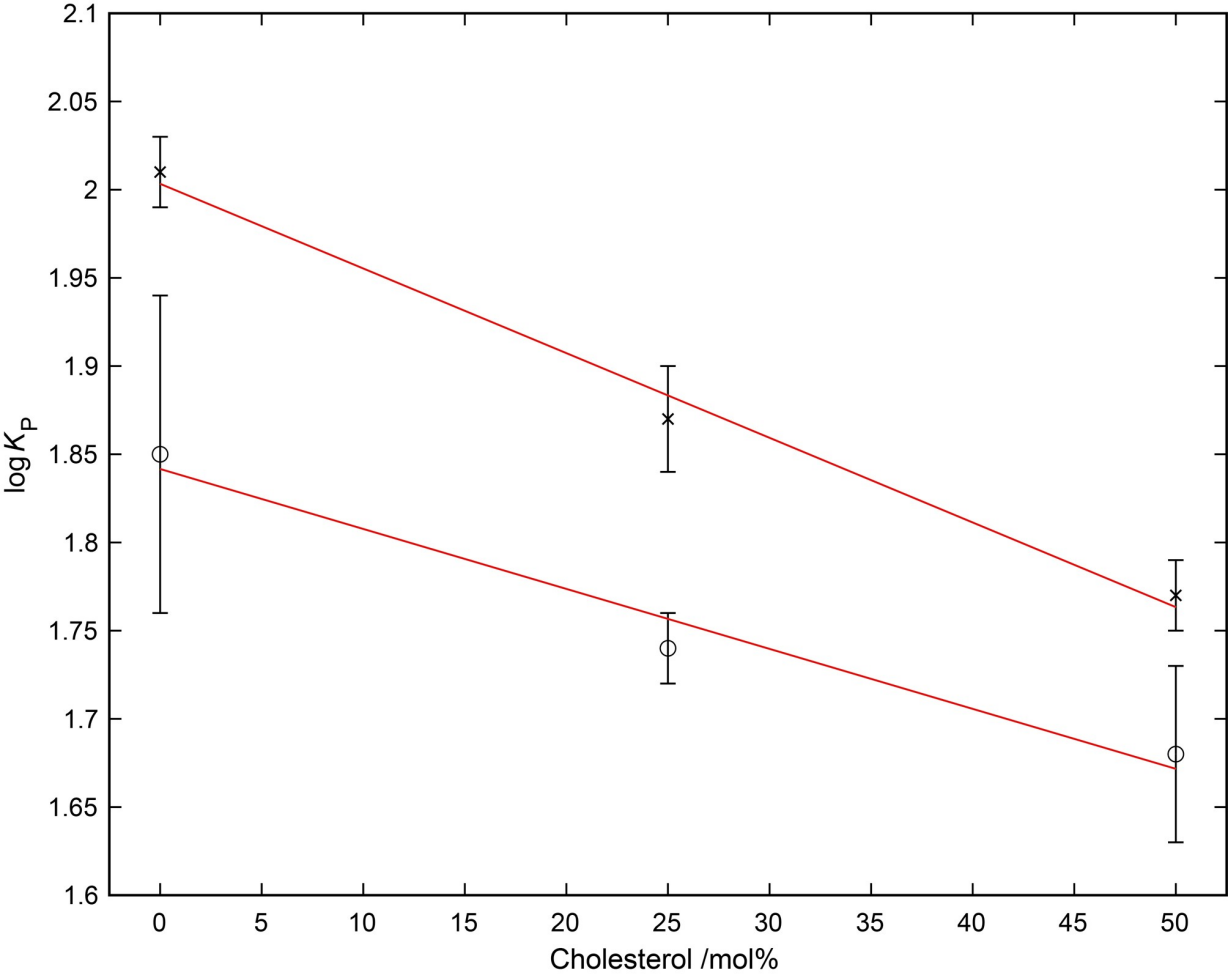
Influence of membrane cholesterol concentration on the partitioning of compounds **9** (x) and **10** (o) into POPC bilayers.

Where the ^19^F chemical shifts of multiple compounds are spectrally resolved, it is also possible to investigate the relative partitioning of compounds in a mixture. Such measurements allow the determination of Δlog*K*_p_ between the compounds studied and are potentially useful if complex lipid mixtures are being studied and the partial molecular volume of the lipid is unknown. To demonstrate this, equimolar mixtures of two pairs of compounds (**9**/**10** and **10**/**13**) were added to multilamellar POPC vesicles. As the compounds in each pair are spectrally well resolved, it is possible to integrate the free and the bound species as described for the individual components and determine the log*K*_p_ directly. Overall, excellent agreement was found between the the log*K*_P_ measured for each compound alone and that measured in equimolar mixtures (Table 1); with both the absolute log*K*_p_ and Δlog*K*_p_ maintained when the compounds were studied in combination. The largest deviation was found for a mixture of compounds **9** and **10** which at 3.3 mol % resulted in a increase in log*K*_p_ of 0.2 units for both species. Interestingly, a reduction in the concentration to 1.65 mol %, such that the overall concentration mirrored those used to obtain log*K*_P_ in isolation, resulted in closer agreement.

**Table 1:** Summary of molar partition coefficients determined from studies containing mixtures of fluorinated substrates. Partitioning studied in POPC bilayers hydrated at 20 % w/v for a given compound to lipid ratio.

log*K*_p_ Measured as equimolar mixture			log*K*_p_ Measured as single compound (3.30 mol%)
**9+10**	1.65 mol% each	1.99±0.09 (**9**) 1.84±0.01 (**10**)	2.01±0.02 (**9**) 1.83±0.04 (**10**)
	3.30 mol% each	2.18±0.07 (**9**) 2.01±0.04 (**10**)	
**10+13**	3.30 mol% each	1.88±0.11 (**10**) 2.50±0.10 (**13**)	1.83±0.11 (**10**) 2.40±0.07 (**13**)

In conclusion, it has been demonstrated that for different sets of fluorinated aliphatics, the changes in log*P*_OW_ arising from variation in fluorination motif are also observed in their respective water‐membrane partition coefficients (log*K*_p_). However, excellent correlations were only obtained if each series is considered separately, which is attributed to steric effects arising from molecules entering the lipid environment. It was interesting to note that for some families of isomers which exhibited little variation in log*P*, greater discrimination was observed in log*K*_p_. An efficient and convenient experimental solid state MAS ^19^F NMR protocol was developed that permits the accurate determination of the molar partition coefficient of fluorinated compounds in membranes of arbitrary complexity was described. This technique exploits the exquisite sensitivity of the ^19^F chemical shift to the local electrostatic environment, far greater than that experienced by protons or deuterons, to allow the resolution of the free and membrane bound fluorinated compounds. Given the sensitivity of current spectrometers, this provides access to the study of log*K*_p_ across approx. 5 units. Our results will be of interest in drug discovery optimisation programmes, as our results clearly indicate that log*P*_OW_ modulation via fluorination is translated in concomitant membrane permeability changes. Furthermore, from the perspective of membrane biophysics, these methods enable us to ascertain how the composition and physicochemical properties of the membrane modulate the partitioning of fluorinated compounds, providing opportunities to tailor fluorination motifs that favour partitioning into particular classes of membranes.

## Conflict of interest

The authors declare no conflict of interest.

## Supporting information

As a service to our authors and readers, this journal provides supporting information supplied by the authors. Such materials are peer reviewed and may be re‐organized for online delivery, but are not copy‐edited or typeset. Technical support issues arising from supporting information (other than missing files) should be addressed to the authors.

Supporting Information

## Data Availability

The data that support the findings of this study are openly available in ePrints from the University of Southampton's research repository (https://doi.org/10.5258/SOTON/D2579).

## References

[1] M. J. Waring , Expert Opin. Drug Discovery 2010, 5, 235–248. 10.1517/17460441003605098 22823020

[2a] J. A. Arnott , S. L. Planey , Expert Opin. Drug Discovery 2012, 7, 863–875; 10.1517/17460441.2012.714363 22992175

[2b] P. D. Leeson , S. A. St-Gallay , Nat. Rev. Drug Discovery 2011, 10, 749–765. 21959288 10.1038/nrd3552

[3a] S. Ritter , W. H. Hauthal , G. Maurer , J. Chem. Eng. Data 1994, 39, 414–417;

[3b] A. Kaune , M. Knorrenschild , A. Kettrup , Fresenius J. Anal. Chem. 1995, 352, 303–312.

[4a] G. P. van Balen , C. Martinet , G. Caron , G. Bouchard , M. Reist , P. A. Carrupt , R. Fruttero , A. Gasco , B. Testa , Med. Res. Rev. 2004, 24, 299–324; 14994366 10.1002/med.10063

[4b] A. Avdeef , Absorption and Drug Development , 2nd ed., Wiley, Hoboken, 2012, pp. 220–250.

[5a] X. Liu , B. Testa , A. Fahr , Pharm. Res. 2011, 28, 962–977; 21052797 10.1007/s11095-010-0303-7

[5b] S. D. Krämer , D. Lombardi , A. Primorac , A. V. Thomae , H. Wunderli-Allenspach , Chem. Biodiversity 2009, 6, 1900–1916. 10.1002/cbdv.200900122 19937828

[6a] R. Welti , L. J. Mullikin , T. Yoshimura , G. M. Helmkamp, Jr. , Biochemistry 1984, 23, 6086–6091; 6525345 10.1021/bi00320a028

[6b] K. Kitamura , M. Yamamoto , S. Takegami , M. Sugiura , Talanta 1999, 49, 261–265; 18967595 10.1016/s0039-9140(98)00384-1

[6c] K. Kitamura , N. Imayoshi , T. Goto , H. Shiro , T. Mano , Y. Nakai , Anal. Chim. Acta 1995, 304, 101–106;

[6d] N. C. Santos , M. Prieto , M. A. Castanho , Biochim. Biophys. Acta Biomembr. 2003, 1612, 123–135. 10.1016/s0005-2736(03)00112-3 12787930

[7a] G. Plemper van Balen , G. Caron , G. Ermondi , A. Pagliara , T. Grandi , G. Bouchard , R. Fruttero , P. A. Carrupt , B. Testa , Pharm. Res. 2001, 18, 694–701; 11465428 10.1023/a:1011049830615

[7b] A. Avdeef , K. J. Box , J. E. A. Comer , C. Hibbert , K. Y. Tam , Pharm. Res. 1998, 15, 209–215; 9523305 10.1023/a:1011954332221

[7c] C. Ottiger , H. Wunderli-Allenspach , Eur. J. Pharm. Sci. 1997, 5, 223–231.

[8] S. D. Kramer , Pharmacokinetic Optimization in Drug Research, Wiley, Hoboken, 2000, pp. 401–428.

[9] B. Testa , P.-A. Carrupt , P. Gaillard , R.-S. Tsai , Lipophilicity in Drug Action and Toxicology, Wiley-VCH, Weinheim, 1996, pp. 49–71.

[10a] S. Saphier , S. Katalan , G. Yacov , A. Berliner , O. Redy-Keisar , G. Fridkin , L. Ghindes-Azaria , I. Columbus , A. Pevzner , E. Drug , H. Prihed , E. Gershonov , Y. Eichen , S. Elias , G. Parvari , Y. Zafrani , Chem. Eur. J. 2023, 29, e202202939; 36374157 10.1002/chem.202202939

[10b] K. P. Melnykov , O. Tavlui , A. Skreminskiy , Y. O. Kuchkovska , O. O. Grygorenko , Chem. Eur. J. 2022, 28, e202201601; 35699162 10.1002/chem.202201601

[10c] Q. Jia , Y. Ni , Z. Liu , X. Gu , Z. Cui , M. Fan , Q. Zhu , Y. Wang , J. Ma , J. Chem. Inf. Model. 2022, 62, 4928–4936; 36223527 10.1021/acs.jcim.2c01201

[10d] S. Holovach , K. P. Melnykov , A. Skreminskiy , M. Herasymchuk , O. Tavlui , D. Aloshyn , P. Borysko , A. B. Rozhenko , S. V. Ryabukhin , D. M. Volochnyuk , O. O. Grygorenko , Chem. Eur. J. 2022, 28, e202200331. 35147261 10.1002/chem.202200331

[11] M. Zhang , T. Peyear , I. Patmanidis , D. V. Greathouse , S. J. Marrink , O. S. Andersen , H. I. Ingólfsson , Biophys. J. 2018, 115, 679–689. 30077334 10.1016/j.bpj.2018.07.010 PMC6104562

[12a] A. A. Omran , K. Kitamura , S. Takegami , M. Kume , M. Yoshida , A. A. Y. El-Sayed , M. H. Mohamed , M. Abdel-Mottaleb , J. Pharm. Biomed. Anal. 2002, 30, 1087–1092; 12408899 10.1016/s0731-7085(02)00438-7

[12b] S. Takegami , K. Kitamura , M. Ohsugi , A. Konishi , T. Kitade , AAPS PharmSciTech 2016, 17, 1500–1506. 26863891 10.1208/s12249-016-0495-1

[13a] S. E. Feller , C. A. Brown , D. T. Nizza , K. Gawrisch , Biophys. J. 2002, 82, 1396–1404; 11867455 10.1016/S0006-3495(02)75494-5 PMC1301941

[13b] H. A. Scheidt , D. Huster , Acta Toxicol. Ther. Acta Pharmacol. Sin. 2008, 29, 35–49; 10.1111/j.1745-7254.2008.00726.x 18158864

[13c] T. Kimura , K. Cheng , K. C. Rice , K. Gawrisch , Biophys. J. 2009, 96, 4916–4924; 19527650 10.1016/j.bpj.2009.03.033 PMC2712040

[13d] D. Ntountaniotis , T. Kellici , A. Tzakos , P. Kolokotroni , T. Tselios , J. Becker-Baldus , C. Glaubitz , S. Lin , A. Makriyannis , T. Mavromoustakos , Biochim. Biophys. Acta Biomembr. 2014, 1838, 2439–2450; 10.1016/j.bbamem.2014.06.003 PMC4321718 24946142

[13e] O. Engberg , H. A. Scheidt , T. K. M. Nyholm , J. P. Slotte , D. Huster , Langmuir 2019, 35, 11902–11911; 31424941 10.1021/acs.langmuir.9b01202

[13f] J. Kremkow , M. Luck , D. Huster , P. Muller , H. A. Scheidt , Biomol. Eng. 2020, 10, 1384. 10.3390/biom10101384 PMC7650631 32998467

[14] D. A. Middleton , D. G. Reid , A. Watts , J. Pharm. Sci. 2004, 93, 507–514. 14705206 10.1002/jps.10544

[15a] S. E. Chambers , E. Y. Lau , J. T. Gerig , J. Am. Chem. Soc. 1994, 116, 3603–3604;

[15b] E. Y. Lau , J. T. Gerig , J. Am. Chem. Soc. 1996, 118, 1194–1200;

[15c] E. Y. Lau , J. T. Gerig , J. Am. Chem. Soc. 2000, 122, 4408–4417.

[16] B. Jeffries , Z. Wang , R. I. Troup , A. Goupille , J.-Y. Le Questel , C. Fallan , J. S. Scott , E. Chiarparin , J. Graton , B. Linclau , Beilstein J. Org. Chem. 2020, 16, 2141–2150. 32952731 10.3762/bjoc.16.182 PMC7476584

[17] R. I. Troup , B. Jeffries , R. E. Saudain , E. Georgiou , J. Fish , J. S. Scott , E. Chiarparin , C. Fallan , B. Linclau , J. Org. Chem. 2021, 86, 1882–1900. 33400526 10.1021/acs.joc.0c02810

[18a] R. Lipowsky , E. Sackmann , Structure and dynamics of membranes: I. from cells to vesicles/II. generic and specific interactions, Elsevier, Amsterdam, 1995;

[18b] G. van Meer , Trends Cell Biol. 1998, 8, 29–33. 9695805 10.1016/s0962-8924(97)01196-3

[19a] K. Korten , T. J. Sommer , K. W. Miller , Biochim. Biophys. Acta Biomembr. 1980, 599, 271–279; 10.1016/0005-2736(80)90073-5 7397149

[19b] R. A. Smith , E. G. Porter , K. W. Miller , Biochim. Biophys. Acta Biomembr. 1981, 645, 327–338; 10.1016/0005-2736(81)90204-2 7272292

[19c] W. K. Subczynski , J. S. Hyde , A. Kusumi , Proc. Natl. Acad. Sci. USA 1989, 86, 4474–4478; 2543978 10.1073/pnas.86.12.4474 PMC287292

[19d] W. K. Subczynski , A. Wisniewska , J.-J. Yin , J. S. Hyde , A. Kusumi , Biochemistry 1994, 33, 7670–7681. 8011634 10.1021/bi00190a022

[20] C. L. Wennberg , D. van der Spoel , J. S. Hub , J. Am. Chem. Soc. 2012, 134, 5351–5361. 22372465 10.1021/ja211929h

