# Supplementary material for: Lipophilicity Modulations by Fluorination Correlate with Membrane Partitioning
Source: Angew Chem Int Ed Engl. 2023 Apr 17;62(21):e202301077. doi: 10.1002/anie.202301077 (PMC10946813; doi:10.1002/anie.202301077)
Supplement: Supplementary file 1 — Supporting Information [file ANIE-62-0-s001.pdf]

Angewandte Chemie
Eine Zeitschrift der Gesellschaft Deutscher Chemiker

Supporting Information

Lipophilicity Modulations by Fluorination Correlate with Membrane Partitioning

Z. Wang, H. R. Felstead, R. I. Troup, B. Linclau, P. T. F. Williamson**

Supplementary Material

Table of Contents

1 Detailed description of the protocol 3

1.1 Sample Preparation 3

1.2 [19]F Magic Angle Spinning (MAS) Solid-State NMR 3

1.3 Data processing 4

1.4 Calculation of the molar membrane partition coefficients 4

2 Influence of exchange between the membrane and aqueous phase on ^{19}F MAS-NMR Lineshape (Figure S1) 5

3 Influence of hydration and concentration on partitioning (Figure S2, Table S1), 6

4 Summary of molar partition coefficients (Table S2). 8

5 Overall correlation between logP_{OW} and logK_P (Figure S3) 9

6 Correlation between logP_{OW} and logK_P (Figure S4) 10

7 Influence of the presence of cholesterol on substrate partitioning (Table S3) 11

8 Synthesis and octanol-water log*P* of the compounds 12

8.1 Synthesis of novel compounds 12

8.1.1 Synthesis of methyl 4,6-dideoxy-4,6-difluoro-β-D-glucopyranoside (**6**) 12

8.1.2 Synthesis of methyl 4,6-dideoxy-4,6-difluoro-β-D-galactopyranoside (**8**) 12

8.2 Lipophilicity determination of the glycosides 13

8.2.1 Methodology 13

8.2.2 Standard NMR settings 14

8.2.3 LogP measurement data 14

8.2.3.1 LogP measurement of methyl 4,6-dideoxy-4,6-difluoro-α-D-glucopyranoside (**5**) 14
8.2.3.2 LogP measurement of methyl 4,6-dideoxy-4,6-difluoro-β-D-glucopyranoside (**6**) 14
8.2.3.3 LogP measurement of methyl 4,6-dideoxy-4,6-difluoro-α-D-galactopyranoside (**7**) 15
8.2.3.4 LogP measurement of methyl 4,6-dideoxy-4,6-difluoro-β-D-galactopyranoside (**8**) 15

9 References 15

1 Detailed description of the protocol

1.1 Sample Preparation

Samples of multilamellar vesicles were prepared from a stock solution of 10 mg mL^{-1} POPC in methanol. The relevant quantity of stock solution was placed under high vacuum overnight to remove the solvent resulting in the formation of a thin film of lipid. To this stock solutions of the compound under study were added in water and the final volume adjusted to obtain the correct level of hydration. Typically, samples contained 8 mg of lipid. The samples were subjected to five cycles of freeze, thaw and mixing resulting in a homogeneous emulsion of multilamellar vesicles. The samples were stored at -20 °C and measured within 1 week of preparation. Typically, about 20 uL of sample were loaded into a 2.5 mm Bruker MAS rotor by centrifugation.

1.2 ^{19}F Magic Angle Spinning (MAS) Solid-State NMR

All NMR studies were conducted on a Bruker Avance III or Neo 850 MHz spectrometer equipped with a 2.5 mm HFX probe. The samples were maintained at 25 °C using a Bruker VT unit, which applies a temperature controlled stream of N_2 gas (~750 L/h) to regulate the sample temperature. This ensured that the lipid vesicles remained in their fluid liquid crystalline phase (T_m of pure POPC ~-3°C[1]). All spectra were recorded using a ^{19}F $\pi/2$ pulse of 3 μs and 10 kHz SPINAL proton decoupling[2] during acquisition at a spinning speed of 10 kHz. Data were acquired with 4096 points with a 50 kHz spectra width centred on the resonances of interest. Due to the extreme sensitivity of ^{19}F chemical shift to variations in temperature, care must be taken to avoid sample heating. Experimentally it was found that further increases in proton decoupling led to no further improvements in linewidth, whilst significantly higher decoupling fields led to asymmetric broadening of the lineshapes due to rf induced heating. Heating from MAS was minimised through the use of smaller diameter rotors (2.5 mm), which induce much smaller frictional heating compared to large rotors (eg 7 mm), as well as lower speed spinning which also minimises frictional heating. In this regime changes in sample temperature due to MAS are likely to be <3 °C[3]. All ^{19}F spectra have been referenced externally to the deuterium signal of deuterated acetone.

The determination of the $\log K_P$ is dependent on the acquisition and analysis of quantitative ^{19}F spectra, with care taken to ensure that two populations are excited evenly and allowed to fully recover between experiments. The perturbation of the chemical shifts arising from the partitioning of the substrate into the membrane is typically small (typically <8 kHz/10 ppm in the samples studied) compared to the radiofrequency field strengeth (65 kHz/81 ppm at this magnetic field) and thus aligning the transmitter with the signals of interest ensures that both membrane-partitioned and free substrate are excited equally. When studying mixtures where chemical shift differences between the two compounds could be significantly larger than the

perturbations observed from partitionining of a coupound into the membrane and comparable to the r.f. field strength then care should be taken to ensure that the two compounds are similarly excitated. Quantitative data also requires the complete relaxation of the nuclei between scans. For the studies reported here, saturation recovery measurements were performed on each system, with spectra then acquired with a recycle delay of five times T_1. It was noted that for the systems studied, the membrane bound substrate typically exhibited shorter T_1 values than that of the substrate in aqueous solution, mirroring the observation made in octanol.[4]

In addition to more general NMR considerations, care must also be exercised to ensure that the signal intensity, particularly that of the membrane bound state, is not influenced by anisotropic interactions that may be present. Due to the molecular dynamics within the lipid bilayer, many of these interactions are already motionally averaged facilitating their removal. The application of low-power (10 kHz) SPINAL-64 decoupling[2] is sufficient to remove the weak scalar and dipolar couplings that are present within the fluid lipid bilayer. Care was taken to minimise heating effects during decoupling which at higher powers resulted in significant linebroadening due to temperature dependent changes in chemical shift. Similarly, data was acquired with both 5 and 10 kHz MAS and no change in isotropic chemical shift was observed suggesting frictional heating from the MAS was minimal. However, increasing the spinning speed from 5 to 10 kHz ensured complete averaging the residual chemical shielding anisotropy and other anisotropic contrbutions to the spectra and the presence of a single isotropic resonance for each population. The absence of any discernable sidebands in spectra acquired at 10 kHz allows the accurate integration of the signal intensity without the need to integrate the entire family of sidebands which would be required for a quantitative analysis. Care should be taken when extending this to other compounds where the anisotropy may larger, either due to a larger chemical shielding anisotropy at the fluorine site studied or the application of higher magnetic as this would complicate the signal integration.

1.3 Data processing

Prior to Fourier Transform, data was processed with 1Hz exponential line broadening and zero filled with 16384 points. The resulting spectra was phased and base line corrected. All processing was performed in Matlab using matNMR[5]. The subsequent resonances where fitted to Lorentzian peaks using custom scripts in Matlab which are available from the authors.

1.4 Calculation of the molar membrane partition coefficients

In the slow exchange limit the molar partition coefficient can then be calculated as:

$$K_P = \frac{n_{s,M}/n_M}{n_{s,W}/n_W} = \frac{C_{s,M/total}}{C_{s,W/total}} \cdot \frac{n_W}{n_M} = \frac{I_{s,M}}{I_{s,W}} \cdot \frac{n_W}{n_M}$$

Where $C_{s,L}$ and $C_{s,M}$ are the concentrations of the substrate in the membrane and aqueous phases respectively and n_s, n_M and n_W are the number of substrate, lipid and water molecules respectively. The ratio $C_{s,M/total}/C_{s,W/total}$ can be determined directly from the ratio of the integrals from the signals arising from the lipidic and aqueous phases (See Figure 2, main paper), whilst the ratio of n_W/n_M represents the ratio of water to lipid molecules in the membrane, the degree of hydration.

2 Influence of exchange between the membrane and aqueous phase on ^{19}F MAS-NMR Lineshape (Figure S1)

The influence of chemical exchange is well characterised in the liquid state[6], however exchange processes could potentially interfere with the MAS averaging required to detect the membrane bound substrate in these experiments. To assess the effect of chemical exchange between the free and membrane bound populations of substrate on the MAS lineshapes the exchange processes has been modelled using Spinach[7] and the spectral features studies over a range of spinning speeds and exchange rates. To simulate the lineshape two equal populations were defined with isotropic chemical shifts differing by 10 ppm. Chemical shielding anisotropy (CSA) was included as an axially symmetric tensor whose anisotropy is 12.5 ppm. Although this does not accurately reflect the size of the static tensor, which can be in the order of 200 ppm in the molecules studied, molecular motion in both the aqueous phase, and to a lesser extent the membrane bound phase, results in a significant dynamic averaging of the CSA. Simulations were conducted at 20 T with 1600 powder points summed for each FID. Analysis of the lineshapes over a range of routinely accessible spinning speeds (5 to 20 kHz) (Supplementary Figure S1.) revealed that over a range of exchange rates from spanning 0 to 128000 s^{-1}, exchange between the free and bound pool did not interfere with the averaging of the CSA by MAS, with changes in the lineshape reflecting those one would expect from chemical exchange on a timescale comparable to or exceeding the frequency separation between the resonances of the two populations.[6]

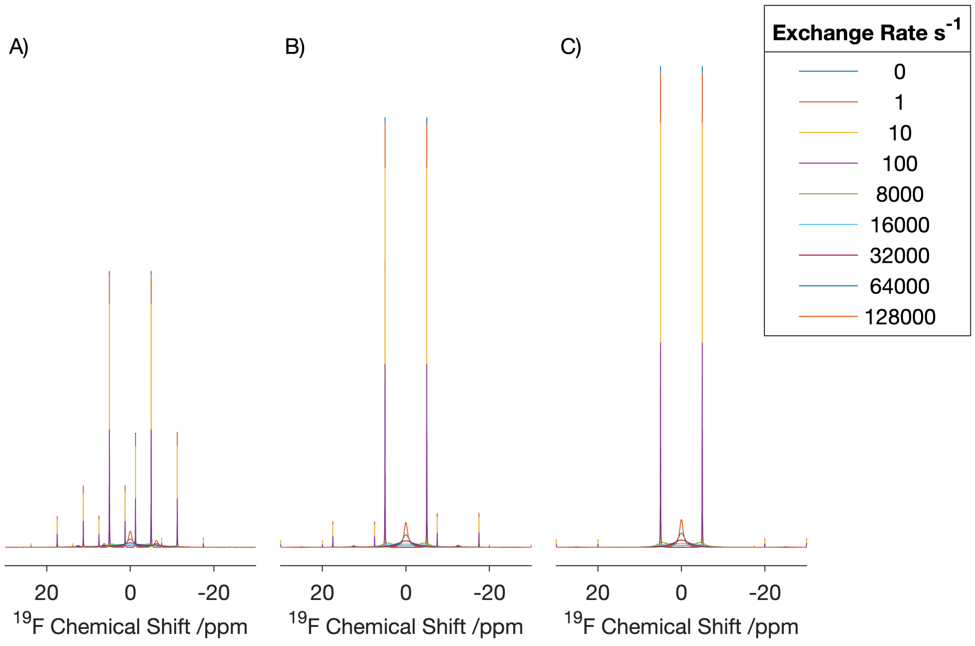

Figure S1. Influence of chemical exchange on MAS lineshapes at 5 (A), 10 (B) and 20 kHz (C) magic angle spinning. Details of simulation parameters are given in the text.

3 Influence of hydration and concentration on partitioning (Figure S2, Table S1),

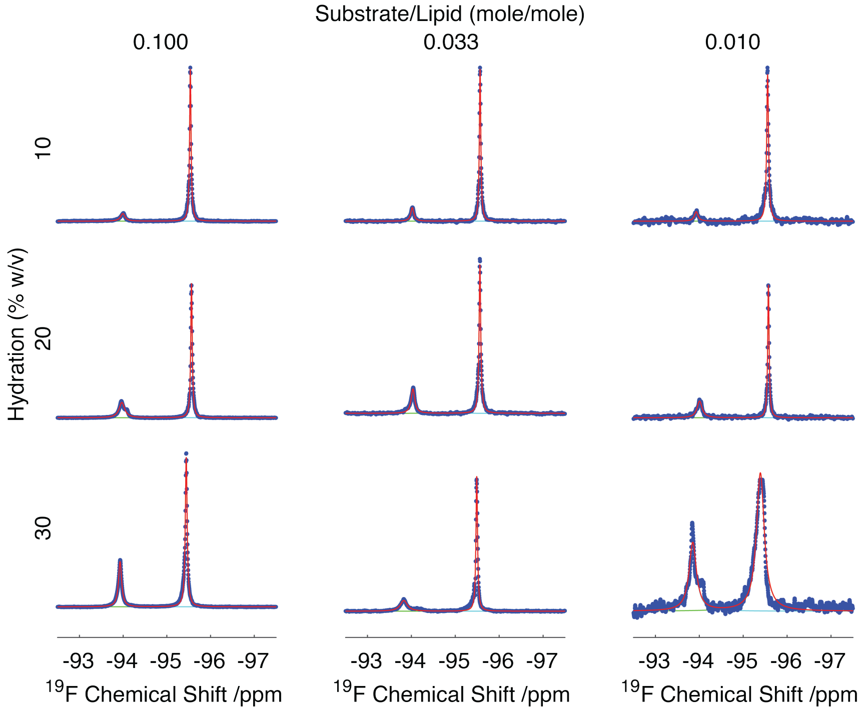

Figure S2. Effect of hydration and substrate/lipid ration on the ^{19}F proton decoupled MAS spectra of compound **10**. Blue points represent experimental data, with Lorentzian fits plotted in red.

Table S1. Influence of hydration and substrate concentration on partition coefficients measured. *Mean value reported (n=3). Values derived from spectra plotted in Figure S3.

Hydration (% w/v)	Substrate/Lipid (mole/mole)	K_P	$\log K_P$
10	0.100	64.48	1.81
10	0.033	81.76	1.91
10	0.010	53.43	1.73
20	0.100	89.41	1.95
20	0.033	67.61	1.83*
20	0.010	93.92	1.97
30	0.100	70.72	1.85
30	0.033	49.23	1.69
30	0.010	65.28	1.81
	Mean	71.57	1.85
	STD	14.37	0.09

4 Summary of molar partition coefficients (Table S2).

	Sample	Molar Partition Coefficient ($\log K_P$)	Free chemical shift (linewidth) /ppm	Bound chemical shift (linewidth) /ppm
**1**	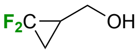	2.54 ± 0.08	-113.75 (0.045)<br>-113.55 (0.058)	-112.88 (0.576)
**2**	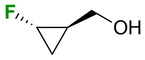	2.17 ± 0.05	-208.95 (0.055)	-209.76 (0.089)
**3**	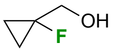	2.08 ± 0.01	-173.96 (0.035)	-173.60 (0.251)
**4**	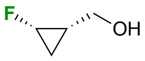	2.02 ± 0.04	-226.36 (0.057)	-226.58 (0.167)
**5**	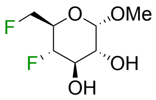	1.98 ± 0.02<br>1.98 ± 0.03	-198.454 (0.030)<br>-216.893 (0.034)	-198.016 (0.078)<br>-216.069 (0.072)
**6**	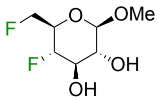	1.93 ± 0.16<br>1.95 ± 0.15	-189.22 (0.050)<br>-224.24 (0.050)	-188.28 (0.117)<br>-222.83 (0.112)
**7**	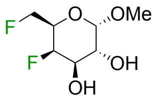	1.80 ± 0.05<br>1.87 ± 0.14	-208.67 (0.031)<br>-214.50 (0.031)	-208.92 (0.126)<br>-214.27 (0.094)
**8**	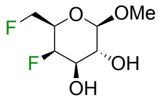	1.69 ± 0.00<br>1.64 ± 0.06	-207.67 (0.029)<br>-214.57 (0.029)	-207.77 (0.063)<br>-214.46 (0.073)
**9**	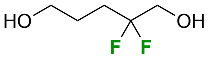	2.01 ± 0.02	-111.823 (0.052)	-110.806 (0.137)
**10**	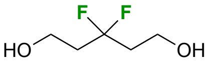	1.83 ± 0.04	-95.56 (0.044)	-94.01 (0.105)
**11**	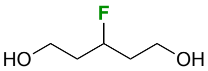	1.68 ± 0.08	-172.19 (0.058)	-172.70 (0.119)
**12**	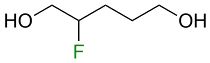	1.45[b]	-175.73 (0.045)	-175.672 (0.046)
**13**	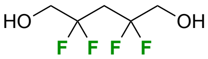	2.40 ± 0.07	-108.80 (0.050)	-108.43 (0.111)
**14**	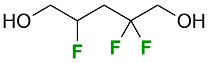	2.01 ± 0.01	-153.15 (0.031)	-152.943 (0.058)
**15**	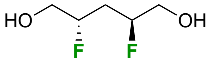	1.85 ± 0.10	-190.26 (0.035)	-190.23 (0.117)
Ent-**15**	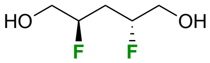	1.82 ± 0.06	-190.35 (0.041)	-190.21 (0.128)
**16**	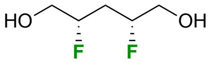	1.71 ± 0.10	-187.90 (-0.032)	-197.42 (0.057)

All samples hydrated at 20% (w/v) with a molar ratio of substrate/lipid of 3.3%. The values reported are the mean and standard deviations measured on three independent samples. a) Where multiple resonances are present, the molar partition coefficient is reported for each site. b) the bound and free resonances could only be resolved in one sample, and the molar partition coefficient is reported for this single measurement.

5 Overall correlation between logP_{OW} and logK_P (Figure S3)

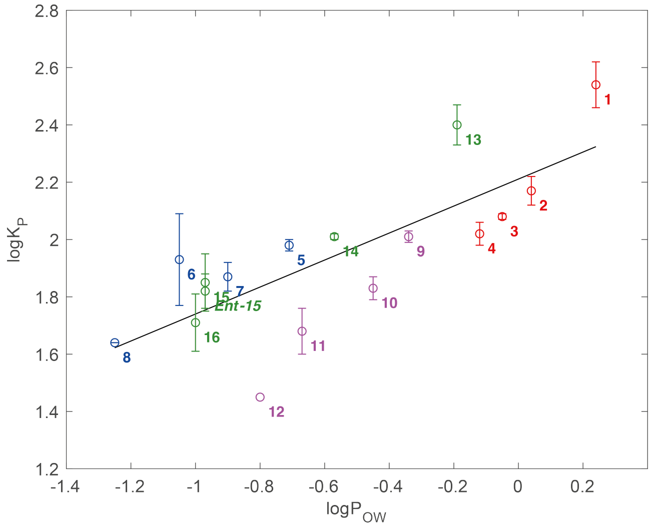

Figure S3. Overall correlation between logP_{OW} and logK_P for all compounds studied, cyclopropyl derivatives. Numbering and logK_P values reported as for Table S2.

6 Correlation between logP_{OW} and logK_P (Figure S4)

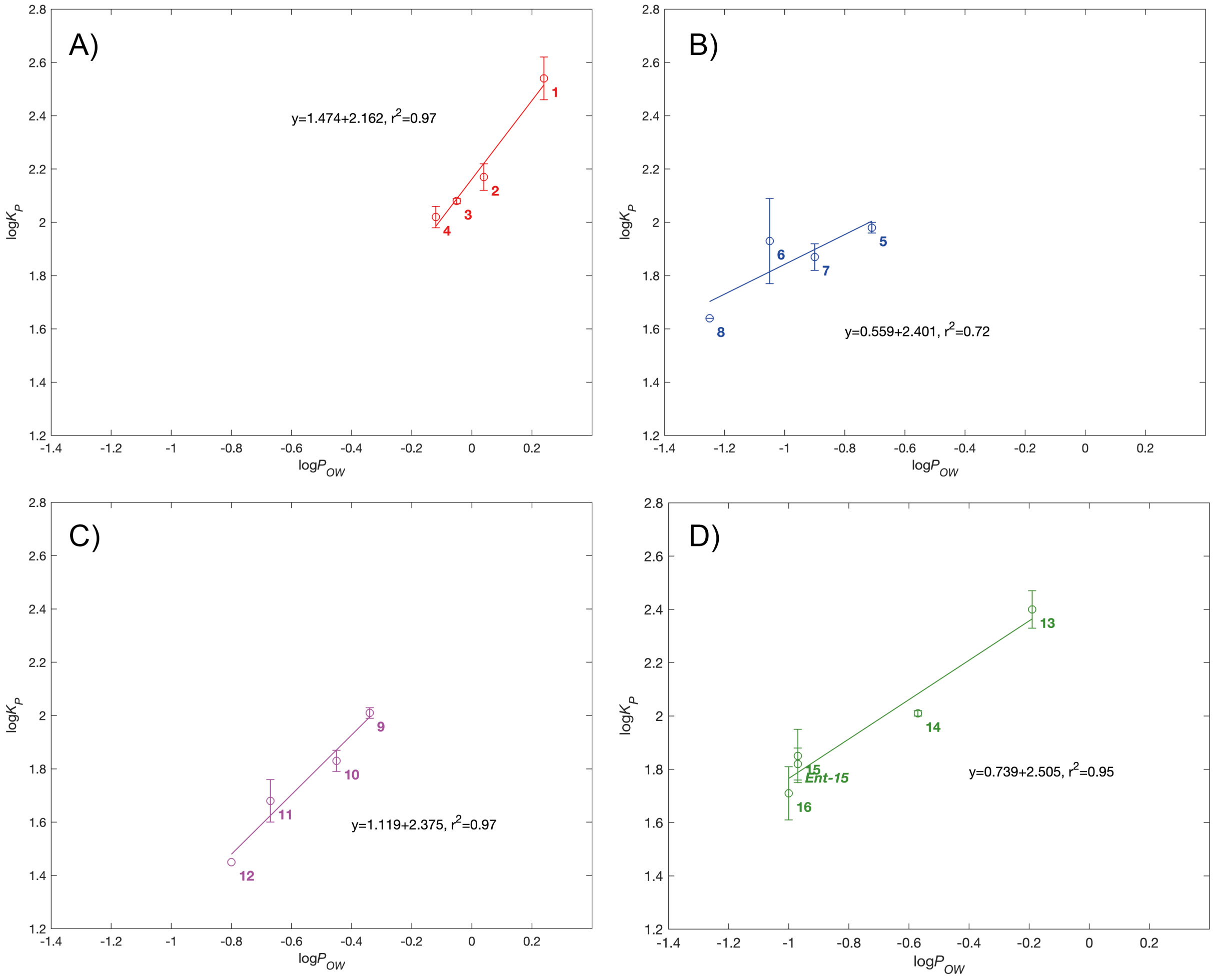

Figure S4. Correlation between logP_{OW} and logK_P for four series of compounds studied, cyclopropyl derivatives (A), glycosides (B), pentane-1,5-diols (single site fluorination, C) and pentane-1,5-diols (skipped fluorination motif, D). Numbering and logK_P values reported as for Table S4.

7 Influence of the presence of cholesterol on substrate partitioning (Table S3)

		Molar Partition Coefficient ($\log K_P$)		
		Membrane Cholesterol Concentration (mol %)		
Sample		0	25	50
9	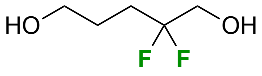	2.01 ± 0.02	1.87 ± 0.03	1.77 ± 0.02
10	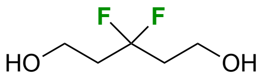	1.85 ± 0.09	1.74 ± 0.02	1.68 ± 0.05

Table S3. Samples prepared with POPC with the appropriate mol % of cholesterol and hydrated at 20% (w/v) with ddH2O containing the substrate at a 3.3 mol% with respect to the POPC concentration. The $\log K_P$ values are reported as the volume per lipid is known to be effected by the presence of cholesterol in the lipid bilayer[8].

8 Synthesis and octanol-water log*P* of the compounds

The synthesis and lipophilicity of the cyclopropylmethyl and the pentane-1,5-diol compounds has been reported.[9] The synthesis of methyl 4,6-dideoxy-4,6-difluoro-α-D-glucopyranoside (**5**)[10] and methyl 4,6-dideoxy-4,6-difluoro-α-D-galactopyranoside (**7**)[10-11] has been described.

8.1 Synthesis of novel compounds

*8.1.1 Synthesis of methyl 4,6-dideoxy-4,6-difluoro-β-D-glucopyranoside (**6**)*

A solution of acetyl chloride (0.3 mL, 4.20 mmol) in anhydrous MeOH (2.5 mL) was stirred at room temperature for 15 min, followed by addition of methyl 4,6-dideoxy-4,6-difluoro-α-D-glucopyranoside **5** (120 mg, 0.61 mmol). The reaction mixture was stirred at 65 °C for 16 h, and then concentrated under reduced pressure. The crude mixture was purified by flash chromatography (acetone/petroleum ether 30/70 – 40/60) to afford a mixture of desired product **6** (calculated yield: 13%) and the starting material **5**. **Selected ^{1}H NMR data** (500 MHz, $CDCl_3$) δ 4.63 - 4.75 (2H, m, H6 + H6', a coupling constant of 47.1 Hz (doublet) for H6 and H6' was observed), 4.41 (1H, ddd, *J* 50.9, 10.1, 8.7 Hz, H-4), 4.28 (1H, d, *J* 7.8 Hz, H-1), 3.82 - 3.86 (1H, m, H-3), 3.63 - 3.71 (1H, m, H-5), 3.59 (3H, s, OC*H*3), 3.44 (1H, t, *J* 8.8 Hz, H-2) ppm; **Selected ^{1}H(^{19}F) NMR data** (500 MHz, $CDCl_3$) δ 4.62 - 4.73 (2H, m, H6 + H6'), 4.41 (1H, dd, *J* 10.0, 8.7 Hz, H-4), 4.28 (1H, d, *J* 7.7 Hz, H-1), 3.86 (1H, t, *J* 9.1 Hz, H-3), 3.66 (1H, ddd, *J* 9.9, 4.5, 1.9 Hz, H-5), 3.59 (3H, s, OC*H*3), 3.44 (1H, dd, *J* 9.3, 7.7 Hz, H-2) ppm; **^{19}F NMR** (376 MHz, $CDCl_3$) δ -200.6 (1F, br. dd, *J* 52.0, 15.6 Hz, F-4), -234.6 (1F, td, *J* 46.8, 24.3 Hz, F-6) ppm; **^{19}F(^{1}H) NMR** (376 MHz, $CDCl_3$) δ -200.6 (1F, s, F-4), -234.6 (1F, s, F-6) ppm; **^{13}C NMR** (101 MHz, $CDCl_3$) δ 103.4 (d, *J* 1.5 Hz, C-1), 87.9 (dd, *J* 183.0, 7.3 Hz, C-4), 81.0 (d, *J* 175.3 Hz, C-6), 74.3 (d, *J* 18.7 Hz, C-3), 73.2 (d, *J* 8.4 Hz, C-2), 72.3 (dd, *J* 24.2, 18.7 Hz, C-5), 57.3 (s, O*C*H3) ppm; **HRMS** (ESI+) for $C_7H_{12}F_2NaO_4$ $(M + Na)^+$ calcd 221.0596, found 221.0598 (-1.0 ppm error).

*8.1.2 Synthesis of methyl 4,6-dideoxy-4,6-difluoro-β-D-galactopyranoside (**8**)*

To a solution of methyl 4,6-dideoxy-4,6-difluoro-α-D-galactopyranoside (**7**) (0.10 g, 0.51 mmol, 1.00 equiv) in MeOH (1.05 mL) was added acetyl chloride (0.16 mL, 2.27 mmol, 4.50 equiv) dropwise at 0 °C under an inert atmosphere. The solution was heated to 65 °C for 16 h. The reaction was cooled, $NaHCO_3$ (0.18 g, 2.52 mmol, 5.00 equiv) was added portionwise and the reaction mixture concentrated. The crude product was purified by via flash column chromatography (SiO_2, CH_2Cl_2/MeOH 0/100 to 5/95). Fractions containing desired product were combined to afford title compound **8** as a white powder (38.0 mg, 0.19 mmol, 62%) as

an inseparable 1.0:0.7 mixture of α:β anomers. **R$_f$** 0.22 (MeOH/CH_2Cl_2 5/95); **IR** (neat) 3350 (br), 2954 (m), 1056 (s), 1026 (s) cm^{-1}; **^{1}H NMR** (500 MHz, MeOD) δ 4.76 (1H, d, *J* = 3.4 Hz, H-1$_α$), 4.76 (1H, dd, *J* = 51.7, 2.3 Hz, H-4$_α$), 4.64 (1H, m, H-4$_β$, overlapped with H-4$_α$ and H-6$_α$ or H-6'$_α$), 4.59 (1H, dddd, *J* = 47.6, 9.4, 7.6, 1.1 Hz, H-6$_β$ or H-6'$_β$), 4.58 (1H, ddd, *J* = 46.0, 10.1, 5.5 Hz, H-6$_α$ or H-6'$_α$), 4.54 (1H, ddd, *J* = 46.2, 9.6, 5.3 Hz, H-6$_β$ or H-6'$_β$), 4.46 (1H, dddd, *J* = 46.7, 9.6, 7.1, 1.1 Hz, H-6$_α$ or H-6'$_α$), 4.22 (1H, dd, *J* = 7.7, 1.0 Hz, H-1$_β$), 4.08 (1H, dddd, *J* = 31.1, 13.7, 7.6, 5.0 Hz, H-5$_α$), 3.92 (1H, ddddd, *J* = 27.5, 12.1, 6.9, 5.5, 0.5 Hz, H-5$_β$), 3.83 (1H, ddd, *J* = 28.4, 10.5, 2.1 Hz, H-3$_α$), 3.76 (1H, ddd, *J* = 10.3, 3.7, 1.8 Hz, H-2$_α$), 3.60 (1H, ddd, *J* = 30.2, 10.3, 2.5 Hz, H-3$_β$), 3.53 (3H, s, H-7$_β$), 3.48 (1H, ddd, *J* = 9.8, 7.8, 1.6 Hz, H-2$_β$), 3.42 (3H, s, H-7$_α$) ppm; **^{13}C NMR** (101 MHz, MeOD) δ 105.8 (s, C-1$_β$), 101.7 (s, C-1$_α$), 91.3 (dd, *J* = 179.7, 5.9 Hz, C-4$_α$), 90.1 (dd, *J* = 181.2, 6.6 Hz, C-4$_β$), 82.9 (dd, *J* = 168.0, 6.6 Hz, C-6$_α$), 82.6 (dd, *J* = 169.4, 5.1 Hz, C-6$_β$), 73.4 (d, *J* = 18.3 Hz, C-3$_β$), 73.2 (dd, *J* = 22.7, 17.6, Hz, C-5$_β$), 72.5 (d, *J* = 1.5 Hz, C-2$_β$), 70.2 (d, *J* = 2.9 Hz, C-2$_α$), 70.0 (d, *J* = 18.3 Hz, C-3$_α$), 69.5 (dd, *J* = 22.0, 17.6 Hz, C-5$_α$), 57.5 (s, C-7$_β$), 56.1 (s, C-7$_α$) ppm;**^{19}F NMR** (376 MHz, MeOD) δ -232.8 (td, *J* = 46.8, 12.1 Hz, F-6$_β$), -232.4 (td, *J* = 46.8, 13.8 Hz, F-6$_α$), -221.5 (dt, *J* = 51.2, 29.0 Hz, F-4$_α$), - 219.1 (dt, *J* = 57.2, 27.7 Hz, F-4$_β$) ppm;**^{19}F(^{1}H) NMR** (376 MHz, MeOD) δ -232.4 (s, F-6$_β$), -232.8 (s, F-6$_α$), -221.5 (s, F-4$_α$), - 219.1 (s, F-4$_β$) ppm; **HRMS** (ESI+) for $C_7H_{12}F_2NaO_4$ (M + Na)$^+$ calcd 221.0597, found 221.0596. (-0.6 ppm error).

8.2 Lipophilicity determination of the glycosides

8.2.1 Methodology

Lipophilicities were determined using a previously published protocol:[4] to a 10 mL pear-shaped flask was added the compound (1.0 - 10 mg) for log*P* determination, the reference compound (1.0 - 10 mg, with known log*P* value, e.g., 2,2,2-trifluoroethanol, log*P*: +0.36), water (2 mL) and *n*-octanol (2 mL). The resulting biphasic mixture was stirred (at 600 rpm) for 2 h at 25 °C, and then left without stirring for 16 h at 25 °C to allow phase separation. An aliquot of 0.5 mL was taken from each phase using 1 mL syringes with long needles and added to two separate NMR tubes. A deuterated NMR solvent (0.1 mL, e.g., acetone-d$_6$), or a capillary tube containing deuterated NMR solvent, was added to the NMR tubes to enable signal locking. Because of the volatility of the used compounds, the NMR tubes were sealed using a blowtorch. For NMR samples with directly added deuterated solvent, the tubes were inverted 20 times for mixing. For ^{19}F(^{1}H) NMR experiments, NMR parameters were set as follows: D1 30 sec for the octanol sample, D1 60 sec for the water sample; and O1P centered between two diagnostic fluorine peaks. If needed, an increased number of transients (NS) and/or narrower spectral window (SW) for a good S/N ratio (typically >300) was applied. After NMR data processing, integration ratios $ρ_{oct}$ and $ρ_{aq}$ ($ρ_{oct}$ is defined as the integration ratio between

the compound and the reference compound in the octanol sample; likewise for ρ_{aq}) were obtained, and used in the equation ($\log P^X = \log P^{ref} + \log(\rho_{oct}/\rho_{aq})$) to obtain the log*P* value of the compound. The log*P* measurement of each compound was run in triplicate. Log*P* values of non-fluorinated compounds were taken from the literature.

The standard deviation (σ) was calculated using the formula:

$$\sigma = \sqrt{\frac{1}{N}\sum_{i=1}^{N}(x_i - \bar{x})^2}$$

Where x_i are the individual estimated log*P* values and $\bar{x}$ is the mean average

8.2.2 Standard NMR settings

Standard NMR parameter setting: SW, 300 ppm; centered O1P; NS 64; D1 30 sec (octanol sample), D1 60 sec (water sample). Any change from standard setting was described for each compound.

8.2.3 LogP measurement data

8.2.3.1 LogP measurement of methyl 4,6-dideoxy-4,6-difluoro-α-D-glucopyranoside (**5**)

Compound (Ref: ZW8456-15)	Nr	Experiments (octanol/water)	ρ_{oct}/ ρ_{wat}	log*P*	Average log*P*	Error
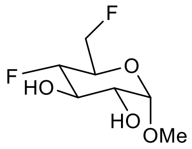	**5**	fe0118zw1/ fe0118zw2	0.4082/ 0.3618	-0.698	-0.71	-0.707 (±0.008)
		fe0118zw4/ fe0118zw5	0.4022/ 0.3649	-0.708		
		fe0118zw6/ fe0118zw7	0.3911/ 0.3626	-0.717		

*Reference compound: 2-fluoroethanol (-0.75).

**Change from standard in NMR parameter setting: SW (120 ppm).

8.2.3.2 LogP measurement of methyl 4,6-dideoxy-4,6-difluoro-β-D-glucopyranoside (**6**)

Compound (Ref: ZW8456-20)	Nr	Experiments (octanol/water)	ρ_{oct}/ ρ_{wat}	log*P*	Average log*P*	Error
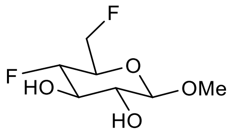	**6**	fe0818zw1/ fe0818zw2	0.0777/ 0.1646	-1.036	-1.05	-1.048 (±0.008)
		fe0818zw3/ fe0818zw4	0.0742/ 0.1638	-1.054		
		fe0818zw5/ fe0818zw6	0.0750/ 0.1652	-1.053		

*Reference compound: methyl 4,6-dideoxy-4,6-difluoro-α-D-glucopyranoside (log*P*: -0.71).

**Change from standard in NMR parameter setting: SW (120 ppm).

8.2.3.3 LogP measurement of methyl 4,6-dideoxy-4,6-difluoro-α-D-galactopyranoside (7)

Compound (Ref: ZW8456-60)	Nr	Experiments (octanol/water)	ρ_{oct}/ ρ_{wat}	log*P*	Average log*P*	Error
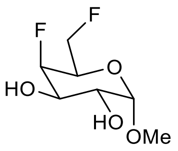	**7**	ju0718zw1/ ju0718zw2	0.2820/ 0.4027	-0.905	-0.90	-0.902 (±0.003)
		ju0718zw3/ ju0718zw4	0.2830/ 0.3980	-0.898		
		ju0718zw5/ ju0718zw6	0.2808/ 0.3982	-0.902		

*Reference compound: 2-fluoroethanol (-0.75).

**Change from standard in NMR parameter setting: SW (120 ppm).

8.2.3.4 LogP measurement of methyl 4,6-dideoxy-4,6-difluoro-β-D-galactopyranoside (8)

Compound (Ref: ZW8456-61)	Nr	Experiments (octanol/water)	ρ_{oct}/ ρ_{wat}	log*P*	Average log*P*	Error
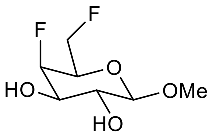	**8**	ju0818zw3/ ju0818zw4	0.3561/ 0.8108	-1.257	-1.25	-1.251 (±0.006)
		ju0818zw11/ ju0818zw12	0.3728/ 0.8196	-1.242		
		ju0818zw13/ ju0818zw14	0.3647/ 0.8219	-1.253		

*Reference compound: methyl 4,6-dideoxy-4,6-difluoro-α-D-galactopyranoside (log*P*: -0.90).

**Change from standard in NMR parameter setting: SW (120 ppm); octanol sample, NS (512).

9 References

[1] B. J. Litman, E. N. Lewis, I. W. Levin, *Biochem.* **1991**, *30*, 313-319.
[2] B. M. Fung, A. K. Khitrin, K. Ermolaev, *J. Magn. Reson.* **2000**, *142*, 97-101.
[3] G. M. Bernard, A. Goyal, M. Miskolzie, R. McKay, Q. Wu, R. E. Wasylishen, V. K. Michaelis, *J. Magn. Reson.* **2017**, *283*, 14-21.
[4] B. Linclau, Z. Wang, G. Compain, V. Paumelle, C. Q. Fontenelle, N. Wells, A. Weymouth-Wilson, *Angew. Chem. Int. Edit.* **2016**, *55*, 674-678.
[5] J. D. van Beek, *J. Magn. Reson.* **2007**, *187*, 19-26.
[6] G. Bodenhausen, R. Ernst, A. Wokaun, *Principles of nuclear magnetic resonance in one and two dimensions*, [Reprinted (with further corrections)] ed., Clarendon Press, Oxford, **1997**.
[7] H. J. Hogben, M. Krzystyniak, G. T. Charnock, P. J. Hore, I. Kuprov, *J. Magn. Reson.* **2011**, *208*, 179-194.

[8] a) M. Alwarawrah, J. Dai, J. Huang, *J. Chem. Theory Comput.* **2012**, *8*, 749-758; b) A. I. Greenwood, S. Tristram-Nagle, J. F. Nagle, *Chem. Phys. Lipids* **2006**, *143*, 1-10.

[9] a) B. Jeffries, Z. Wang, R. I. Troup, A. Goupille, J.-Y. Le Questel, C. Fallan, J. S. Scott, E. Chiarparin, J. Graton, B. Linclau, *Beilstein. J. Org. Chem.* **2020**, *16*, 2141-2150; b) R. I. Troup, B. Jeffries, R. E. Saudain, E. Georgiou, J. Fish, J. S. Scott, E. Chiarparin, C. Fallan, B. Linclau, *J. Org. Chem.* **2021**, *86*, 1882-1900.

[10] D. E. Wheatley, C. Q. Fontenelle, R. Kuppala, R. Szpera, E. L. Briggs, J. B. Vendeville, N. J. Wells, M. E. Light, B. Linclau, *J. Org. Chem.* **2021**, *86*, 7725-7756.

[11] a) C. W. Somawardhana, E. G. Brunngraber, *Carbohydr. Res.* **1981**, *94*, C14-C15; b) C. W. Somawardhana, E. G. Brunngraber, *Carbohydr. Res.* **1983**, *121*, 51-60; c) P. J. Card, G. S. Reddy, *J. Org. Chem.* **1983**, *48*, 4734-4743.
